# Rapid Deployment of a Free, Privacy-Assured COVID-19 Symptom Tracker for Public Safety During Reopening: System Development and Feasibility Study

**DOI:** 10.2196/19399

**Published:** 2020-08-13

**Authors:** Seble G Kassaye, Amanda Blair Spence, Edwin Lau, David M Bridgeland, John Cederholm, Spiros Dimolitsas, JC Smart

**Affiliations:** 1 Department of Medicine Georgetown University Washington, DC United States; 2 LEDR Technologies Inc Seattle, WA United States; 3 Hanging Steel Productions LLC Sterling, VA United States; 4 Office of the Senior Vice President for Research Georgetown University Washington, DC United States

**Keywords:** COVID-19, SARS-CoV-2, home isolation, quarantine, symptom monitoring, information systems, privacy, contact tracing, virus, transmission, public health, eHealth

## Abstract

**Background:**

Since the emergence of severe acute respiratory syndrome coronavirus 2 (SARS-CoV-2), the number of cases of coronavirus disease (COVID-19) in the United States has exponentially increased. Identifying and monitoring individuals with COVID-19 and individuals who have been exposed to the disease is critical to prevent transmission. Traditional contact tracing mechanisms are not structured on the scale needed to address this pandemic. As businesses reopen, institutions and agencies not traditionally engaged in disease prevention are being tasked with ensuring public safety. Systems to support organizations facing these new challenges are critically needed. Most currently available symptom trackers use a direct-to-consumer approach and use personal identifiers, which raises privacy concerns.

**Objective:**

Our aim was to develop a monitoring and reporting system for COVID-19 to support institutions conducting monitoring activities without compromising privacy.

**Methods:**

Our multidisciplinary team designed a symptom tracking system after consultation with experts. The system was designed in the Georgetown University AvesTerra knowledge management environment, which supports data integration and synthesis to identify actionable events and maintain privacy. We conducted a beta test for functionality among consenting Georgetown University medical students.

**Results:**

The symptom tracker system was designed based on guiding principles developed during peer consultations. Institutions are provided access to the system through an efficient onboarding process that uses clickwrap technology to document agreement to limited terms of use to rapidly enable free access. Institutions provide their constituents with a unique identifier to enter data through a web-based user interface to collect vetted symptoms as well as clinical and epidemiologic data. The website also provides individuals with educational information through links to the COVID-19 prevention recommendations from the US Centers for Disease Control and Prevention. Safety features include instructions for people with new or worsening symptoms to seek care. No personal identifiers are collected in the system. The reporter mechanism safeguards data access so that institutions can only access their own data, and it provides institutions with on-demand access to the data entered by their constituents, organized in summary reports that highlight actionable data. Development of the system began on March 15, 2020, and it was launched on March 20, 2020. In the beta test, 48 Georgetown University School of Medicine students or their social contacts entered data into the system from March 31 to April 5, 2020. One of the 48 users (2%) reported active COVID-19 infection and had no symptoms by the end of the monitoring period. No other participants reported symptoms. Only data with the unique entity identifier for our beta test were generated in our summary reports.

**Conclusions:**

This system harnesses insights into privacy and data sharing to avoid regulatory and legal hurdles to rapid adaption by entities tasked with maintaining public safety. Our pilot study demonstrated feasibility and ease of use. Refinements based on feedback from early adapters included release of a Spanish language version. These systems provide technological advances to complement the traditional contact tracing and digital tracing applications being implemented to limit SARS-CoV-2 transmission during reopening.

## Introduction

The emergence of severe acute respiratory syndrome coronavirus 2 (SARS-CoV-2) and the resulting coronavirus disease (COVID-19) pandemic have led to significant morbidity and mortality worldwide, with over 9,473,214 confirmed infections and 484,249 deaths by June 26, 2020 [1]. The first travel-related case of COVID-19 in the United States was reported to the US Centers for Disease Control and Prevention (CDC) on January 21, 2020; the United States has since contributed the majority of COVID-19 cases globally, with 2,367,064 cases and 121,645 deaths reported by June 26, 2020 [1]. This has led to tremendous strain on institutions and agencies working to treat infection and prevent viral transmission [2,3]. Most jurisdictions implemented social distancing and stay-at-home orders; these mitigation strategies have been applied with positive effect in multiple settings [4,5]. In the United States, the daily rate of new diagnoses of infection peaked on April 22, 2020, with 31,994 cases reported on April 12, 2020, then declined to below 14,000 cases daily by mid-May; this rate then began to rise again as stay-at-home restrictions were lifted based on federal guidance, with almost 40,000 cases reported on June 24, 2020 [6-8]. Multidimensional approaches to limit SARS-CoV-2 transmission are critical during the reopening of educational, social, and business entities.

COVID-19 causes a spectrum of disease severity, and up to one-fifth of individuals with COVID-19 infection develop severe disease that requires hospitalization [6,9,10]. Critical components to address the SARS-CoV-2 pandemic and break transmission chains is to identify infected individuals through testing, isolate those with infection, and perform contact tracing to identify and quarantine individuals exposed to active COVID-19 cases. Most individuals with COVID-19 do not require hospitalization; monitoring people who have the illness under home isolation is important to detect persistent or worsening disease that may warrant evaluation. In addition, individuals who have been exposed require quarantine during the potential incubation period; also, based on current guidance, individuals exposed to COVID-19 cases should be monitored in home quarantine for up to 14 days to limit transmission during the asymptomatic or pre-symptomatic period, when transmission may also occur [11,12].

These routine public health strategies limit community spread of infection. However, traditional contact tracing mechanisms in the context of the SARS-CoV-2 pandemic with large numbers of cases in the setting of low community immunity requires great resources [13]. Monitoring a multitude of individuals can quickly exceed institutional capacity, and traditional contact tracing lacks the required speed to identify and reach contacts at high risk of becoming infected and transmitting infection [14]. Given the scale of the response required, technological advances can complement traditional contact tracing methods to introduce efficiencies needed to successfully avert ongoing transmission [15]. Digital contact tracing using real-time locator systems, including downloadable apps, has gained traction as a direct-to-consumer approach to efficiently identify individuals who may have come into close contact with persons diagnosed with COVID-19 [16,17]. These systems require broad community acceptance and usage to provide sufficient population-wide coverage, as mathematical modelling estimates suggest that high population coverage is needed to effectively reduce transmission [18,19]. These data further suggest that a combination of direct contact tracing with a digital approach has the highest yield in identifying cases that warrant isolation or quarantine to successfully mitigate ongoing transmission [16]. However, digital privacy remains a concern, and limited voluntary use of contact tracing approaches hampers the utility of these systems [20].

Our multidisciplinary team has implemented systems using Georgetown University’s AvesTerra framework for privacy-assured technology for HIV surveillance [21-23]. We then sought to design a user-friendly system to efficiently track symptoms associated with COVID-19 infection to complement existing and evolving contact tracing approaches. We conducted a beta test of the symptom tracker to determine the usability of the system and reporter and evaluate the system’s functionality to provide institutions and agencies with summary reports to identify individuals with changing health status during isolation or quarantine.

## Methods

### Background

We identified the need for a symptom tracking system after consultation with experts responding to the evolving pandemic in metropolitan Washington, DC. The overarching purpose was to provide institutions and agencies that were tasked with tracking and monitoring a set constituency with technology-based options that could accommodate exponential increases in use in the event of large-scale COVID-19 outbreaks. We established a multidisciplinary team with expertise in clinical infectious diseases, epidemiology and public health, computer science and systems development, ethics and privacy, and organizational strategy. We identified key data elements that are important for COVID-19 tracking, including epidemiology and exposure, clinical signs and symptoms, risk factors for severe disease, and SARS-CoV-2 testing and results, based on emerging reports and scientific literature at the time of development [24-26].

The system was designed in the Georgetown University AvesTerra knowledge management environment, which supports integration and synthesis of data to identify actionable events. The proposed use, design, and content of the tracker were reviewed by the University General Counsel to guide the development of the Terms of Use and the modality whereby users interface with the system. The design considerations included the following features to increase the usability and acceptability of the system. The system is directed to institutions and agencies to provide access to their populations using unique identifiers known only to the originating institute or agency; no personal identifiers are collected, which limits regulatory hurdles and personal inhibitions to using the system; the Terms of Use are streamlined to avoid legal barriers and the need for arduous and time-consuming data sharing agreement processes; immediate and on-demand access is provided to reports by institutions and agencies reflecting data collected from their population only, with built-in safeguards that limit their access to data from their own population; the development team is willing to customize the system to accommodate the unique needs of individual institutions and agencies; and the system is scalable to millions of users.

We beta-tested the COVID-19 Symptom Tracker under a protocol deemed exempt by the Georgetown University Institutional Review Board. Georgetown University medical students were invited to participate by email, with a link to a Qualtrics survey used to describe the project, provide instructions, and document consent. A random unique ID number was directly generated in Qualtrics for each consenting individual. Participants were asked to enter data twice daily for 3 days. The research team downloaded an aggregate summary report. No personal identifiers were available to the study team.

### Target Audience

The system is directed to institutions and agencies that are tasked with monitoring individuals in home isolation or quarantine. Participating institutions are provided with a link to a page outlining the Terms of Use (Multimedia Appendix 1). These Terms of Use describe the intended use of the system and provide guidance on accessing the system, with emphasis on ensuring collection of deidentified data. The system uses a clickwrap agreement to indicate acknowledgement of the Terms of Use. Once enrolled, the institution can provide their selected constituent population with access to the system. Institutions are assigned a unique 5-digit institution code. The institution is instructed to provide each person entering data with a unique identifier using the 5-digit prefix followed by 6 additional digits. The originating institution maintains the link between the assigned unique identifier and the individual who is being asked to enter symptom data. With this design, no personal identifiers are collected in the system. Participating institutions and agencies are provided with a Reporter executable file and unique authorization code to access data linked to their own entity. The institutions and agencies maintain access to the unique identifiers assigned to the individuals they ask to enter data into the system, thereby maintaining privacy and confidentiality with respect to our development team and other system users. The system is designed to accommodate data from millions of unique individuals.

### Symptom Selection

We selected the two most common symptoms that were reported in early large population–based epidemiology studies: fever (88% to 89%) and cough (68% to 72%) [25]. We selected two additional symptoms that had lower and variable incidence in different publications that we deemed important to capture based on relative frequency or as a potential indicator of disease severity: shortness of breath (18.7%), and sore throat (13.9%) [24]. The option to include additional symptoms using free text was also included in the database design to allow for future iterations and adaptations depending on frequency of reporting.

### Exposure, Epidemiology, and Risk Data

We included elements that were deemed critical to risk-stratify individuals for disease based on emerging epidemiology. The symptom tracker collects data on known exposures to individuals with COVID-19, whether in the household or in a health care setting. Information about potential work exposure is gathered, such as whether the user is a health care worker, first responder, or in other at-risk categories. International and domestic travel history is also elicited. Information on underlying health conditions that are associated with worse COVID-19 outcomes is collected; the individual is prompted to enter a categorical response (yes or no) to whether they have any comorbidities of concern. These comorbidities include underlying hypertension, cardiovascular disease, pulmonary disease, and primary or secondary immunodeficiency.

### End User Safety

The user interface was designed to provide educational information and links to published COVID-19 prevention recommendations from the CDC. The documentation on the user interface also clearly directs individuals who are entering symptom data to seek additional care if they have new signs or symptoms suggestive of incident or worsening infection.

### System Reports

Institutions were provided with an 8-digit authorization code linked to their 5-digit institution identifier during initial enrollment. Agencies and institutions can automatically generate reports that include only their institution-specific data. Actionable information such as the ID number alert for a person with new symptoms is provided to guide institutional decision making and need for follow-up with the individual, who may require testing. Other information provided in the reports includes cessation of symptoms and duration of monitoring, which may trigger removal from isolation for individuals who no longer have symptoms or have met the minimum required period of quarantine. Information on individuals who have not submitted data for the past 24 hours is also generated, which allows institutions to identify subgroups of individuals who may need to be contacted to prompt continued engagement and entry of symptoms into the system or to determine whether the individual has worsening symptoms that may have led to hospitalization or that warrant referral.

### Privacy

Privacy considerations were a critical design element. As the COVID-19 Symptom Tracker is not a direct-to-consumer app, the enrolled institutions and agencies were instructed to provide unique identifiers that were not linkable to individuals. Only enrolled institutions can provide unique identifiers, which contain the prefix assigned to that institution. Thus, only the enrolled institution or agency knows the identity of an individual user. The system provides instructions to individuals to not include or upload personal identifiers when entering data. In addition, periodic scans of uploaded data are performed to ensure that possible personal identifiers are not being entered in the sections that allow free text, and unstructured data that fit the format of a phone number or date of birth are terminally deleted from the free text sections. Deidentified data entered by an individual are only accessible in reports to the institution that provided the user identifier to the individual, thus protecting the data from access by other institutions or agencies. The development team provided a clear statement about the intended use of the data in the Terms of Use, which is accessible to both institutions and users for review. The text was written in simple language with a twelfth grade readability score using the Flesch Kincaid Grade tool. Data can be released to an institution for users who entered data using the institution’s unique 5-digit code. Otherwise, data will only be released if required under subpoena or another legal process.

## Results

### Symptom Tracker System

**Figure 1:**
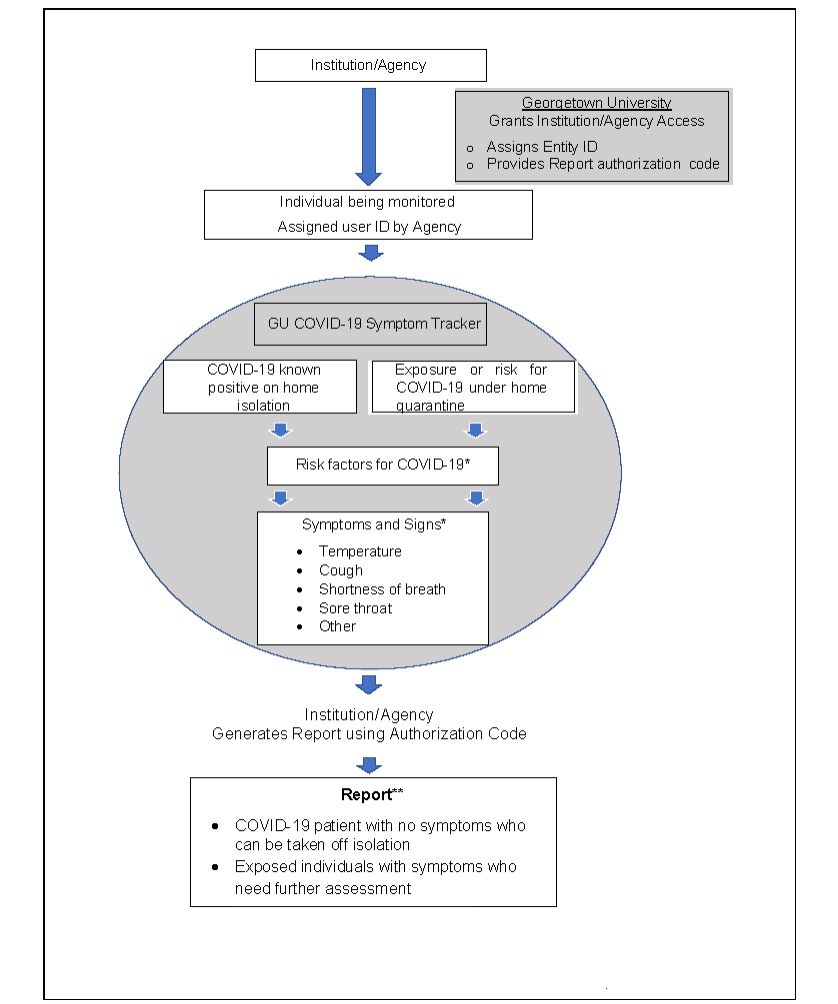
Schema of enrollment in and use of the COVID-19 Symptom Tracker system. COVID-19: coronavirus disease. GU: Georgetown University. *Individuals enter data without personal identifiers based on instructions from the institution or agency. The instructions provided on the website direct individuals with new or worsening symptoms to contact their health care providers. **Institutions and agencies can determine the frequency at which they generate reports.

The symptom tracker system was designed to encompass functionality to ensure privacy without compromising utility while fulfilling the core guiding principles that were developed during peer consultations. We incorporated several distinguishing features into the system design to increase the usability and acceptability of the system; these are summarized in Figure 1. System development began on March 15, 2020, and the symptom tracker was launched on March 20, 2020 [27]; subsequent iterative improvements were made based on requests from early adapters (including a Spanish language version).

### System Beta Test

A total of 48 users from the Georgetown University School of Medicine participated in beta testing conducted between March 31 and April 5, 2020 (Table 1). One of the 48 users (2%) reported active COVID-19 infection, and 47 individuals (98%) were not infected. On the last day of monitoring, the individual with COVID-19 infection was asymptomatic. None of the 47 other participants reported symptoms of COVID-19 infection. By the end of follow-up, 38 of the 47 individuals (81%) had completed three days of data uploads.

**Table 1:** Report from the beta test from March 31 to April 5, 2020 obtained at 5:49 PM on April 5 (N=48).

Characteristic		Value (%)
**COVID-19^a^** **infection, n (%)**		
	Infected	1 (2)
	Infected with temperature lower than 99.3 ºF^b^	1 (2)
	Infected with no cough	1 (2)
	Infected with no shortness of breath	1 (2)
	Infected without data in last 24 hours	0 (0)
**Exposure, n (%)**		
	Exposed but not known to be infected	47 (98)
	Exposed with temperature greater than 99.5 ºF	0 (0)
	Exposed with temperature greater than 100.0 ºF	0 (0)
	Exposed with cough	0 (0)
	Exposed with shortness of breath	0 (0)
	Exposed without data in the last 24 hours	38 (79)
**Infection source, n (%)**		
	Infected, exposed by close contact	0 (0)
	Infected, exposed by health care worker	1 (2)
	Health care workers infected	1 (2)
**Infected users, ID number^c^**		
	Infected with temperature lower than 99.3 ºF	GUTST_001111
	Infected with no cough	GUTST_001111
	Infected with no shortness of breath	GUTST_001111

^a^COVID-19: coronavirus disease.

^b^ºF: degrees Fahrenheit.

^c^ID numbers have been altered for publication purposes.

## Discussion

### Principal Findings

The first diagnosed case of COVID-19 infection in Washington, DC was associated with hundreds of potentially exposed individuals who were required to self-quarantine in early March. The patient was a church rector who revealed his COVID-19 infection status to his congregation and the media. At least five individuals from the rector’s parish subsequently tested positive for COVID-19 [28,29]. This case demonstrated the emerging public health challenges and demands on contact tracing that would result as the pandemic unfolded, and it was a driving motivator in the development of this system for use by institutions and agencies tasked with monitoring individuals under home isolation or quarantine. The resulting product, which we have since deployed, is a user-friendly and scalable rapid response system to efficiently monitor individuals who have or have been exposed to COVID-19 while maintaining their privacy.

Our product was intended to support public health agencies and occupational health teams. With this in mind, we incorporated several distinguishing features from existing products into the system design to increase the usability and acceptability of the system. We developed a streamlined onboarding process and designed a system that would not collect personal identifiers, as standard procedures to execute data use agreements that are required when identifiers are included are arduous and impractical during public health emergencies. As health agencies are potential intended users, we included a link to the recently introduced limited waiver of Health Insurance Portability and Accountability Act (HIPAA) sanctions that were passed in the context of the SARS-CoV-2 pandemic in case these concerns would result in institutional reluctance to use the system [30]. The overall design and reassurances in the Terms of Use thereby limit regulatory hurdles to usage by institutions and agencies that are typically bound by privacy regulations related to health data.

The lack of collection of identifying data in the system should also reassure individual users and alleviate personal inhibitions that appear to be the main weak point limiting the success of other digital contact tracing apps that require identifying information to be functional. Voluntarism is an important component of the success of these technologies, and the individual entering data must be willing to use the system. In the United States, surveys have found age differences in willingness to share SARS-CoV-2 testing results, ranging from 28% of people aged 18-29 years to 63% of people over 65 years of age [31]. However, a much lower percentage of people (50%) were willing to download an app that would alert them upon detecting proximity to a COVID-19 case, and even fewer (45%) were willing to download an app if their data were to be used by public health professionals for disease tracking [31]. These findings suggest that individuals are swayed in their willingness to use contact tracing apps based on who manages their data, with the highest confidence in data residing with and used by health departments [31]. In our design, unlike most other direct-to-consumer models, a specific institution or agency needs to request use of the system by the individual. Given potential sensitivities around monitoring, it is important to engage communities and populations to support the use of these monitoring and tracking systems and to provide guaranteed protections for users. This ethical guidance on best practices for the use of digital contact tracing and symptom monitoring is evolving and should be considered in the design and implementation phase of technology-based contact tracing adjuncts [32].

Contact tracing and monitoring has previously rested firmly in the realm of public health agencies. However, the current situation has documented outbreaks across a swath of occupations [33,34]. With the phased reopening strategy that is currently being rolled out across the United States, the number of cases is rising [7]. It is becoming increasingly apparent that institutions will need to engage in active monitoring for signs and symptoms of COVID-19 as part of their business practice to prevent local outbreaks. These data suggest that implementation of tracing and monitoring systems will be more acceptable when used in the context of reopening to reinvigorate businesses and support employment opportunities [31]. Thus, it is also important to obtain buy-in from employers and small businesses that may choose to use technologically advanced systems such as the system we designed to conduct local active monitoring to ensure the safety of their employees and customers. As this system is available for free, immediate cost would not be a concern; however, use of the system would require introduction of processes to implement it and track responses that are entered by personnel. Given the high burden of COVID-19 among minority populations, we have also provided a Spanish language version to ensure access to this important demographic [35].

Our beta test demonstrated the usability of the Georgetown University COVID-19 Symptom Tracker. We requested a limited duration of reporting as part of this beta test, primarily to ensure functionality of the reporting system as envisioned. Students responded rapidly when asked to participate, and most of them (38/47, 81%) completed three days of symptom updates. Very few of the medical students reported symptoms; this is likely due to the relative youth and low risk of the students, who were distance-learning from their homes. It is also notable that among over 800 students in the School of Medicine, only 48 chose to participate. This demonstrates potential challenges to voluntary usage of this or any other digital contact tracing or monitoring approach. Because this beta test was portrayed to the students as a feasibility study, the lack of uptake is not generalizable to a scenario where the students would be asked to participate in this monitoring system as a condition to safely restart in-person academic instruction in the fall. Our initial beta testing had limited scope to demonstrate feasibility prior to the rapid deployment of the system. Additional in-depth end user feedback to ensure accessibility, ease of use, and willingness and durability of engagement will also be important to assess. Targeted instruction of thought leaders and the target community are needed to ensure understanding of the scope and purpose of the monitoring system to address any reservations and promote use of the system as a social responsibility for public safety and the greater good of the community.

### Limitations

The selected symptoms were based on reports of hospitalized patients in the early part of the pandemic [24,25]. Since that time, additional symptoms have been recognized as being associated with COVID-19. While the symptom tracker in its current form does not explicitly ask about every possible symptom that we now associate with COVID-19 infection, the most frequent and clinically important symptoms are represented. This limitation can be easily addressed, as the database enables reporting of additional symptoms; in time, this information can be used to guide iterative changes to the database to capture true incident symptoms as they emerge. Our design also decreases the likelihood of user fatigue in entering data that would be further exacerbated if the list of symptoms was further extended, given the limited yield and additionality of symptoms beyond those we selected guided by the literature.

The system currently requires internet access to enter data using the web interface. Having this system available on smartphones and mobile devices using downloadable apps would increase access and improve functionality and flexibility. This may be key to ensuring durable use of the system to maximize effectiveness. Additional customizable prompts (eg, reminders to enter symptoms at a selected frequency) and alerts can also be built in to provide immediate feedback to individuals and to the institution to improve the current functionality and safety features.

### Conclusions

Symptom monitoring systems such as the one we devised and made available for all to use provide technological solutions to support contact tracing and safe reopening in the context of the SARS-CoV-2 pandemic. Privacy issues are addressed, as no personal identifiers are collected. Important health and epidemiologic data are gathered, and the use of these data for purposes of public health and safety is legally permissible and supported. As institutions assume responsibility for monitoring symptoms to protect their constituents, we provide a technological solution to promote efficiency without compromising privacy. With such transparency and assurances, hurdles to large-scale symptom monitoring could be obviated and allow for increased public safety in concert with large-scale contact tracing activities that are already underway. Georgetown University is now applying this privacy-assured technology to an anonymous automated contact tracing system. Public health agencies and occupational health programs should consider using this or another such system as an adjunct to traditional or novel contact tracing approaches to improve efficiency in the race to contain this burgeoning pandemic. We are providing free access to this system, which is scalable to millions of users, to support institutions and organizations within the global community who need to engage in symptom monitoring for public safety.

## Appendix

Multimedia Appendix 1 Georgetown University COVID-19 Symptom Tracker Terms of Use.

## Abbreviations

CDC: US Centers for Disease Control and Prevention
COVID-19: coronavirus disease
HIPAA: Health Insurance Portability and Accountability Act
SARS-CoV-2: severe acute respiratory syndrome coronavirus 2
