# Supplementary material for: Rapid Deployment of a Free, Privacy-Assured COVID-19 Symptom Tracker for Public Safety During Reopening: System Development and Feasibility Study
Source: JMIR Public Health Surveill. 2020 Aug 13;6(3):e19399. doi: 10.2196/19399 (PMC7431234; doi:10.2196/19399)
Supplement: Multimedia Appendix 1 [file publichealth_v6i3e19399_app1.docx]

1. The Tracker is a free online tool provided to User by Georgetown University.  These Terms of Use govern the relationship between User and Georgetown University with respect to use of the Tracker.  User agrees that it has read carefully, understands and agrees to these Terms of Use, and that by using the Tracker User agrees to be legally bound by these Terms of Use.  If User does not agree to these Terms of Use, then User should not use the Tracker.
2. The Tracker is provided on an “as is” basis.  User may use the Tracker at User's sole risk.  Georgetown University makes no representations or warranties concerning the availability of the Tracker or the information contained in the Tracker, and specifically disclaims all such warranties, whether express or implied, including but not limited to the implied warranties of merchantability and fitness for a particular purpose.
3. The Tracker is intended for use by agencies and institutions authorized by Georgetown University for the purpose of monitoring persons on home isolation and/or quarantine.
4. User agrees that it will not enter into the Tracker the personal identifying information of any person.  Instead, User will assign unique identifiers to each person who is instructed by User to enter information into the Tracker.
5. User will use the Tracker to request reports only for persons associated with User by using the five digit identification number assigned to User upon execution of this agreement.
6. User may use the Tracker to monitor the condition of persons on home isolation or quarantine for the purpose of delivering healthcare or public health services or advice.  User understands and agrees that Georgetown University shall not monitor, and shall have no responsibility for monitoring, information contained in the Tracker or taking any action whatsoever based upon that information.
7. User shall be solely responsible for obtaining from persons instructed by User to enter information into the Tracker the full and informed consent of such persons to the collection and use of their information in the Tracker in the manner and for the purposes described in these Terms of Use.
8. User understands and agrees that Georgetown University, at any time and for any reason, may modify or discontinue, temporarily or permanently, the availability of the Tracker, in whole or in part, without notice to User.  User agrees that Georgetown University shall not be liable to User, or to any third party, for any modification or discontinued availability of Tracker.
9. User agrees that it will use the Tracker in compliance all applicable federal, state, local and international laws and regulations, and not for any illegal purpose.
10. User acknowledges that Georgetown University may be required by subpoena or other legal process to provide information contained in the Tracker to third parties, including public health authorities tasked with protecting the public.
11. User agrees to acknowledge its use of the Tracker database when publishing reports or manuscripts prepared using or on the basis of information collected or contained in the Tracker.  Suggested citation is as follows:  “Computational options to vanquish infectious diseases, Georgetown University, COVIDGU.org” all rights reserved 2020
